# MiR-661 promotes tumor invasion and metastasis by directly inhibiting RB1 in non small cell lung cancer

**DOI:** 10.1186/s12943-017-0698-4

**Published:** 2017-07-17

**Authors:** Feiye Liu, Yanjun Cai, Xiaoxiang Rong, Jinzhang Chen, Dayong Zheng, Lu Chen, Junyi Zhang, Rongcheng Luo, Peng Zhao, Jian Ruan

**Affiliations:** 1 Cancer Center, Traditional Chinese Medicine-Integrated Hospital, Southern medical University, Guangdong, 510315 China; 2 Center for Geriatrics, General Hospital of Guangzhou Military Command of People’s Liberation Army, Guangdong, 510010 China; 3 Department of Oncology, Nanfang Hospital, Southern medical University, Guangdong, 510515 China; 4 Department of Medical Oncology, The First Affiliated Hospital, School of Medicine, Zhejiang University, Zhejiang, 310003 China

**Keywords:** miR-661, Non small cell lung cancer, RB, E2F1, Metastasis

## Abstract

**Background:**

Aberrant microRNA expression has been implicated in metastasis of cancers. MiR-661 accelerates proliferation and invasion of breast cancer and ovarian cancer, while impedes that of glioma. Its role in non small cell lung cancer (NSCLC) and underlying mechanism are worthy elucidation.

**Methods:**

Expression of miR-661 was measured with real-time PCR in both NSCLC tissues and cell lines. The effects of miR-661 on migration, invasion and metastasis capacity of NSCLC were evaluated using wound healing, transwell assay and animal models. Dual reporter luciferase assay and complementary experiments were performed to validate RB1 as a direct target of miR-661 for participation in the progression of NSCLC.

**Results:**

MiR-661 was upregulated in NSCLC tissues as compared to paired adjacent tissues and associated with shorter overall survival. Furthermore, miR-661 promoted proliferation, migration and metastasis of NSCLC. Then, we identified RB1 as a direct target of miR-661 through which miR-661 affected EMT process and metastasis of NSCLC. RB1 interacted with E2F1 and both could mediate EMT process in NSCLC.

**Conclusion:**

MiR-661 promotes metastasis of NSCLC through RB/E2F1 signaling and EMT events, thus may serves as a negative prognostic factor and possible target for treatment of NSCLC patient.

**Electronic supplementary material:**

The online version of this article (doi:10.1186/s12943-017-0698-4) contains supplementary material, which is available to authorized users.

## Background

MicroRNAs are 20–22 nt long non-protein-coding RNAs which involve in diverse physiological processes such as proliferation, apoptosis and differentiation [1]. They function as tumor suppressors or oncogenes by promoting degradation or inhibiting translation of regulatory factors through interactions at 3′ UTRs [2]. It is well known that miRNAs are involved in the growth of lung, breast, colorectal and liver cancer by targeting oncogenes like *RAS*, *MYC* and *PTEN* [3]. The functions of miRNAs in metastasis are also worthy attentions. A multitude of miRNAs contribute to metastasis of malignancies including non small cell lung cancer (NSCLC). MiR-132, miR-200 and miR-638 expression are related to inhibition of epithelial–mesenchymal transition (EMT) and metastasis of NSCLC through targeting ZEB2, DLC1, ATRX and SOX2 [4–6], while miR-26a and miR-483-5p accelerates EMT by modulating AKT, RhoGDI1 and ALCAM [7, 8].

MiR-661 was firstly found in breast cancer as a tumor suppressor which inhibits proliferation, motility and invasion of tumor cells through suppression of a metastasis-related protein MTA1 [9]. But another report yielded opposite results that miR-661 accelerates EMT and metastasis of breast cancer cells [10]. Later research elucidated this seemly contradictory phenomenon with the view that miR-661 acts as tumor suppressor in cells with wild type *p53* and oncogene in cells with mutated *p53* [11]. Moreover, miR-661 upregulated in ovarian cancer cells while downregulated in glioma cells [12, 13]. Obviously, miR-661 carries out various function in a tissue-specific and cell-specific pattern. Its role in oncogenesis and metastasis of NSCLC has not been investigated yet.

With E2F1 binding and deactivation, RB1 finely tunes genes involved in cell cycle progression, apoptosis, chromosome stability and cellular metabolism, which results in inhibition of cell proliferation in the development of malignancy [14, 15]. Moreover, angiogenesis and stemness were connected to RB/E2F pathway [16]. Except for this major interaction, RB1 also performs as tumor suppressor through E2F-independent pathways [17–19]. Aberrant RB1 expression are known to promote occurrence of lung cancer [20], but the relationship with invasion process like EMT and underlying mechanism are not well interpreted yet.

In this study, we found that miR-661 was upregulated in NSCLC tissues as compared to adjacent tissues. Overexpressed miR-661 correlated with shorter survival of NSCLC patients. MiR-661 boosted cell proliferation, colony formation accompanied by decreased G1 phase. Further more, elevated miR-661 resulted in enhanced migration and invasion in NSCLC cell lines A549 and SPC-A1. RB1 was a direct target of miR-661 and capable of interacting with E2F1 to enhanced EMT process.

## Methods

### Patients and specimens

Fifty cases of paired NSCLC samples were collected at Nanfang hospital and Cancer Center of Southern medical university (SMUCC). 202 FFPE sections from patients with pathologically confirmed NSCLC who never received pretreatment before radical surgery between 2013 and 2015, were also enrolled in this study. Follow-up information was obtained by telephone or from the outpatient records. The study was approved by Research Ethics Committee of Nanfang Hospital and SMUCC. All samples were used according to the ethical guidelines of the 1975 Declaration of Helsinki and obtained with the patients’ understanding that it might be published.

### Cell culture

All cell lines used in this study were purchased from The Cell Bank of Type Culture Collection of Chinese Academy of Sciences. Human lung epithelial cell EBAS-2B was maintained with RPMI 1640 with 10% FBS. Lung cancer cell lines were cultured with DMEM with 10% FBS. RPMI 1640, DMEM and FBS were purchased from Invitrogen.

### miRNA transfection, siRNA interference and infection

Cells at confluency of 40–50% were transfected with 5 μg of miRNAs using Lipofectamine 2000 reagent (Invitrogen; Carlsbad, Calif, USA). The miR-661 mimic, a nonspecific miR control, anti-miR-661, a nonspecific anti-miR control, lenti-virus with miR-661 and a correspondent control were purchased from GenePharma (Shanghai, China). SiRNA against RB1 and control siRNA were used as Table 1.

**Table 1:** Sequences of human *RB1* shRNAs

No.	shRNA Targets of *RB1*
siRNA-RB1-2	GGGUUGUGUCGAAAUUGGATT
siRNA-RB1-4	GGAGUAUGAUUCUAUUAUATT
Negative control siRNA (NC siRNA)	UUCUCCGAACGUGUCACGUTT

### Real-time PCR

Total RNAs were extracted using Trizol reagent (Invitrogen, Carlsbad, CA, USA)according to the manufacturer’s instructions. 1 μg of total RNA was used to reverse transcribe cDNA with SuperScript® III First-Strand Synthesis System (Invitrogen, Carlsbad, CA). Real-time PCR was performed using ABI 7500HT system. PCR reactions were carried out in a final volume of 10 uL of mixture consisting of SYBR green 2X master mixture (Invitrogen, Carlsbad, CA, USA). The primer sequences were as Table 2, U6 was used as an internal control.

**Table 2:** Oligo Sequences for Real-time PCR

No.	Sequence
hsa-miR-661	Forward: 5′-ACACTCCAGCTGGGTGCCTGGGTCTCTGGCCT-3′ Reverse: 5′- CTCAACTGGTGTCGTGGA-3′
U6	Forward: 5′- CTCGCTTCGGGCAGCACA-3′ Reverse: 5′- AACGCTTCACGAATTTGCGT-3′
RB1	Forward: 5′- GAACATCGAATCATGGAATCCCT-3′ Reverse: 5′- AGAGGACAAGCAGATTCAAGGTGAT-3′
E2F1	Forward: 5′- AGATGGTTATGGTGATCAAAGCC-3′ Reverse: 5′- ATCTGAAAGTTCTCCGAAGAGTCC-3′

### Wound healing and transwell assay

Cells at 80–90% confluency were maintained in serum-free medium for 6 days and then plated in six-well plates containing serum-free medium 24 h before experiments. 10 μl pipette tips were used to scratch constant-diameter stripes in the confluent monolayers. The wounds were photographed at 0, 24, 48 h under an inverted phase contrast microscope. Three random fields were marked and measured. Migration index was expressed by ratio of migrating distance of treated cells to those of control cells.

For transwell assay, cells were treated with mitomycin before seeding in the upper chambers of 24-well transwell plates (Corning Incorporated, New York, NY, USA) pre-coated with 50% matrigel (BD Biosciences, Franklin, New Jersey, USA) in phosphate-buffered saline. The lower chambers were filled with culture medium supplemented with 10% FBS. After incubation for 24 h, invaded cells on the bottom were fixed and stained with 0.5% crystal violet and visualized under a bright field microscopy (OLYMPUScx31, TOKYO, Japan).

### Dual reporter luciferase assay

MiRwalk database (http://zmf.umm.uni-heidelberg.de/apps/zmf/mirwalk2/) were used to screen for direct targets of miR-661. The 1.8 kb full-length of RB1 3′ untranslated region (3′UTR) was cloned into psiCHECKTM-2 dual luciferase reporter vector (Promega; Madison, Wis, USA) and named wild-type (wt) 3′UTR. Site-directed mutagenesis of the miR-661 binding site in the RB1 3′UTR was carried out using the GeneTailor Site-Directed Mutagenesis System (Invitrogen) and named mutant (mt) 3′UTR. Cells were seeded in 96-well culture plates (Costar) and transfected with wt or mt 3′UTR construct along with miR-661 mimic or inhibitor in triplicate. Firefly and renilla luciferase activities were measured at 48 h after cotransfection with the Dual-Luciferase Reporter Assay kit (Promega, Madison, Wis, USA).

### Western blot

Protein expression was assessed by immunoblot analysis of cell lysates (20–60 μg) in RIPA buffer in the presence of rabbit antibodies to E-cadherin, mouse antibodies to β-catenin, fibronectin, vimentin, β-actin (1:500; Santa Cruz, California, USA); and rabbit antibodies to RB1, GAPDH, E2F1, (1:1000; CST, Danvers, MA). Quantifications were carried out using Scion Image software and normalized to β-actin levels.

### Co-immunoprecipitation

Cells were lysed in RIPA buffer with PMSF and proteinase inhibitor for 60 min. Supernatants were collected and incubated with anti-RB1 or anti-E2F1 magnetic beads (biotool. China) for overnight at 4 °C, followed by washing with binding buffer and denaturation in elution buffer. Resuspending and elution. Aliquots of the products were subjected to Western blot analysis as described above.

### Animal models

For lung metastasis model, 5*10^6^ cells of each group were injected into the caudal vein of 10 BalB/c-nu mice (Laboratory Animal Center of Southern Medical University, Guangzhou, China). Animals were sacrificed 60 days after injection. Lung tissues were fixed in formalin and sectioned for H&E staining.

For subcutaneous model, 1*10^6^ cells of each group were injected into the flank region of 4-week old BalB/c-nu mice. Five weeks later, animals were sacrificed and tumor were collected and sectioned for immunohistochemistry.

The experiments on mice had been approved by the ethics committee at SMUCC (Laboratory Animal Center of Southern Medical University, Guangzhou, China).

### Immunohistochemistry

3–4 μm thick tissue sections were subsequently deparaffinized, rehydrated and rinsed before antigen retrieval and endogenous peroxidase blocking. Following non specific binding blocking with goat serum, the slides were incubated with anti-RB1 (1:50; Abcam, Cambridge, UK), E2F1 (1:50; Abcam, Cambridge, UK), vimentin (1:50; Santa Cruz, California, USA) and E-cadherin (1:50; Santa Cruz, California, USA) primary antibody at 4 °C. Standard DAB method (Beyotime, China) was employed to detect the staining. Counter staining was carried out with hematoxylin.

### Immunofluorescence

Cells were cultured on coverslips overnight, fixed with 4% paraformaldehyde and treated with 0.25% Triton X-100. After blocking in 10% normal blocking serum, slides were incubated with rabbit antibodies to E-cadherin, mouse antibodies to β-catenin, vimentin (1:500; Santa Cruz, California, USA), and rabbit antibodies to RB1 and E2F1 (1:100; Abcam, Cambridge, UK) antibodies at 4 °C overnight followed by washing with PBS three times. Cover slips were then incubated with fluorescein isothiocyanate (FITC)-conjugated anti-rabbit or mouse and Texas Red (TR)-conjugated anti-mouse or rabbit antibodies (1:120; SantaCruz) and stained with 6-diamidino-2-phenylindole (DAPI; Invitrogen).

### Statistical analysis

Statistical analyses were performed using a statistical software package (SPSS19.0, Chicago, IL). The results from real-time PCR, immunoprecipitation, dual reporter luciferase assay, wound healing assay and transwell assay were determined by independent t-test. Chi-square tests were conducted to assess correlations between miR-661 expression and clinicalpathological factors. Overall survivals were evaluated using the Kaplan and Meier survival curves, and compared by the log-rank test. Numbers of metastatic lung nodules in mice were compared with independent t-test. Pearson′s correlation analysis was carried out to determine the relation between miR-661 and RB1 as well as E2F1 expression. *P*-value less than or equal to 5% were considered statistically significant.

## Results

### MiR-661 expression was upregulated in NSCLC and an indicator for poorer prognosis

**Fig. 1:**
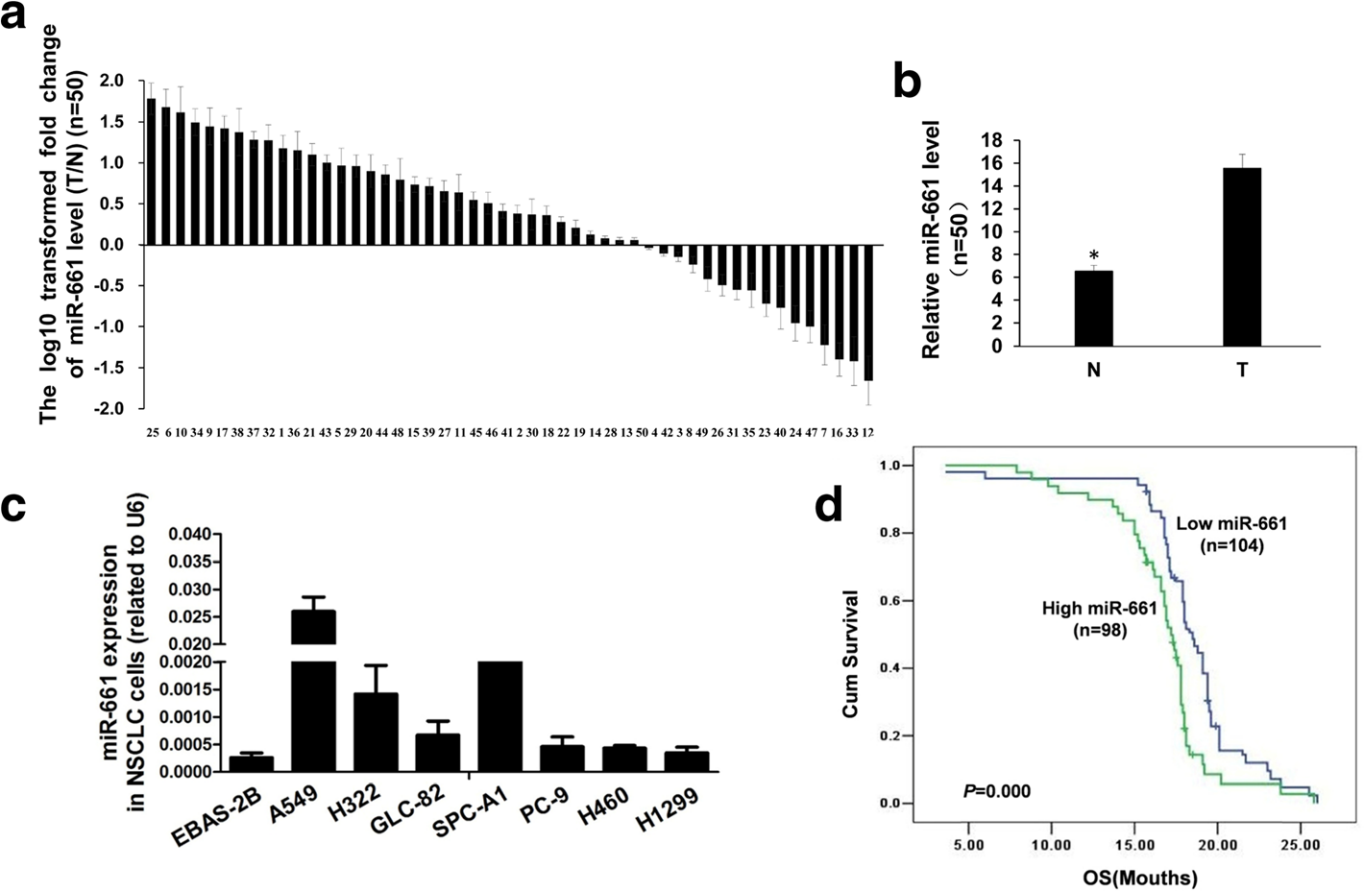
MiR-661 was upregulated in NSCLC and associated with poor prognosis in NSCLC patients. QRT-PCR was used to measure miR-661 expression in human samples and lung cancer cell lines. **a**. Relative miR-661 levels in 50 cases of NSCLC tumors and adjacent tissues. **b**. Comparison of miR-661 expression between NSCLC cancerous and adjacent tissues. **c**. MiR-661 expression in 7 NSCLC cell lines, with normal lung epithelial cell line EBAS-2B as control. **d**. Kaplan-Meier analysis of overall survivals between NSCLC patients with high (*n* = 98) and low (*n* = 104) miR-661 expression. Error bars represent mean (*n* = 3) ± SEM. (* *P* < 0.05)

To measure miR-661 expression in NSCLC tissue, real-time PCR was performed in 50 NSCLC specimens and paired adjacent tissues. We found that miR-661 expression was elevated in 68% (34/50) lung cancer tissues as compared with corresponding adjacent tissues (Fig. 1a). Consistently, relative miR-661 level in NSCLC tissues was significantly higher than adjacent tissue (Fig. 1b), suggesting that miR-661 was overexpressed in NSCLC tissues. Next, enhanced miR-661 expression was also observed in seven NSCLC cell lines comparing to normal human bronchial epithelium cell line EBAS-2B (Fig. 1c). A549 and SPC-A1 expressed the highest level of miR-661 and were chosen for further study. Furthermore, miR-661 expression were significantly correlated with tumor size, lymph node and metastasis status instead of age, gender, smoking status, histologic type or EGFR mutation status (Table 3). Patients with high miR-661 expression (*n* = 98, HR = 2.246, 95% CI 1.619-3.116) suffered from shorter overall time than those with low miR-661 expression (*n* = 104, *P* < 0.000) (Fig. 1d). Therefore, miR-661 expression may predict for unfavorable prognosis of NSCLC patients.

**Table 3:** Correlations between miR-661 and clinical pathological factors

Factors	Tissue miR-661 (*n* = 202)		*P*
	Low (*n* = 104)	High (*n* = 98)	
Age (years)			0.566
≤ 65	26	28	
>65	78	70	
Gender			0.844
Male	46	42	
Female	58	56	
Smoking Status			0.871
Nonsmoker	50	46	
Ever-smoker	54	52	
Histologic Type			0.615
Adenocarcinoma	40	36	
Squamous	52	46	
Others	12	16	
T status			0.003
T1-2	70	46	
T3-4	34	52	
N status			0.003
N0-1	78	54	
N2-3	26	44	
M status			0.001
M0	80	54	
M1	24	44	
EGFR mutation status			0.071
Mutated	40	26	
Wild Type	64	72	

### MiR-661 promotes migration, invasion and metastasis of NSCLC

We further investigate whether miR-661 also function in the development of NSCLC except for serving as a prognostic factor. CCK8 assay (Additional file 1: Figure S1A) and colony formation assay (Additional file 1: Figure S1C) showed that upregulated miR-661 resulted in significantly faster cell growth (*P* < 0.01) and more colony formations (*P* < 0.01) in A549 and SPC-A1, while decreased miR-661 expression had opposite effects. Decreased portion of G1 phase was observed in A549 and SPC-A1 with miR-661 overexpression as compared with control cell lines (both *P* < 0.01) (Additional file 1: Figure S1B). Consistent results were also obtained in nude mice that A549 carrying ectopic miR-661 formed larger (Additional file 1: Figure S1D) and heavier tumor at a faster speed than control group (Additional file 1: Figure S1E).

**Fig. 2:**
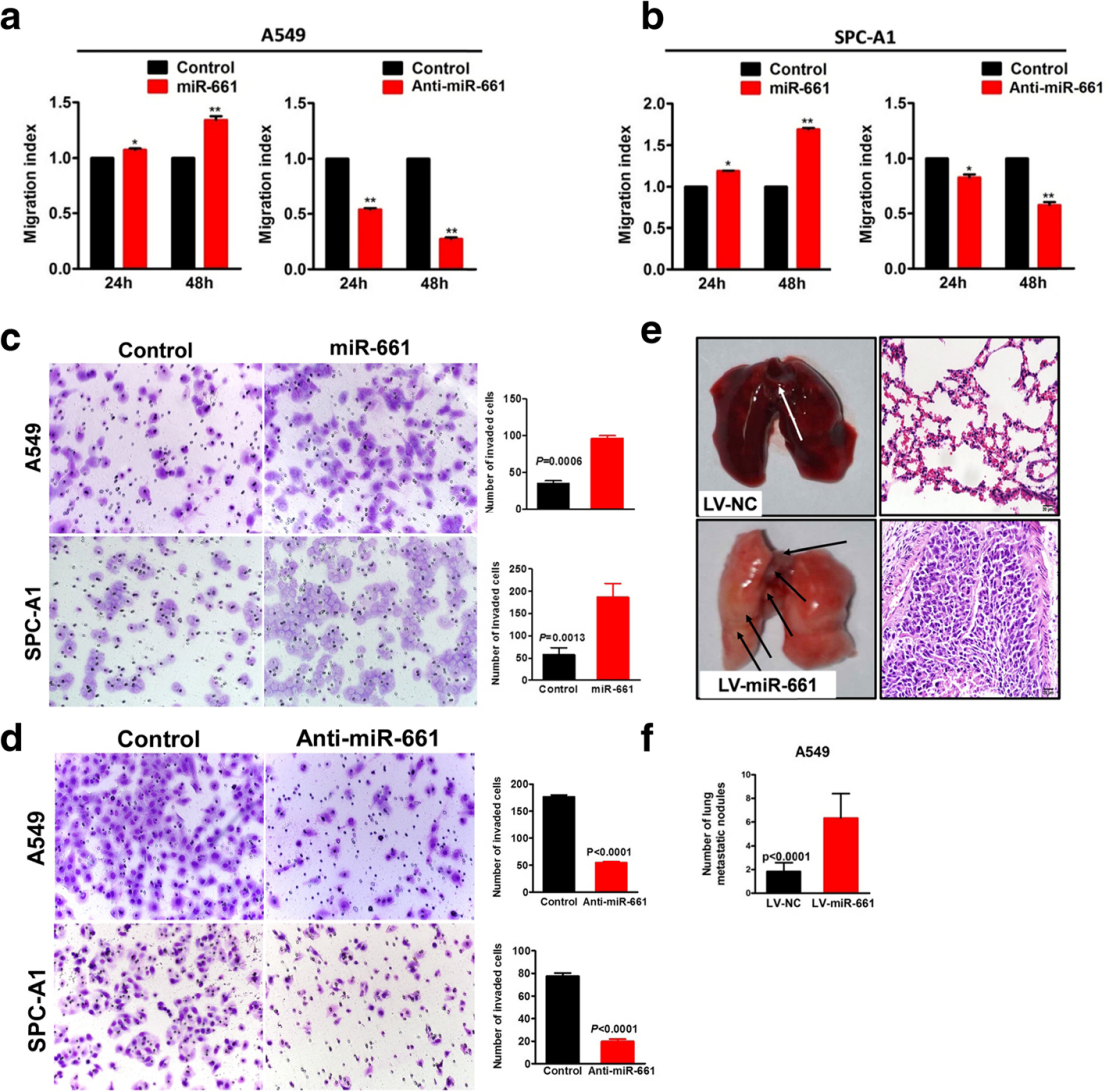
MiR-661 promoted invasion and metastasis of NSCLC. **a**. Wound healing assay of A549 with miR-661 overexpression or anti-miR-661 expression at 0 h, 24 h and 48 h after transfection. Migration distance (μm) was calculated by subtracting the distance at 24 h or 48 h from that at 0 h. Migration ability was expressed by migration index-the distance migrated by treated cells relative to that by control cells. **b**. Wound healing assay of SPC-A1 with miR-661 overexpression or anti-miR-661 expression at 24 h and 48 h after transfection. Migration ability was expressed by migration index. **c**. Transwell assay of A549 and SPC-A1 with increased miR-661 expression. **d**. Transwell assay of A549 and SPC-A1 with miR-661 inhibitor (anti-miR-661). **e**. H&E staining of lung nodules formed after injection of A549 carrying miR-661 (LV-miR-661) and empty vector (LV-NC) through tail vein. **f**. Comparison of numbers of metastatic nodules formed after caudal vein injection of A549 carrying LV-miR-661 and LV-NC. All experiments were performed in triplicates. Error bars are mean (*n* = 3) ± SEM (* *P* < 0.05, ** *P* < 0.01) in panel **a, b, c** and **d**

Besides, wound healing assay indicated that miR-661 upregulation was accompanied by enhanced migration of A549 (Fig. 2a) and SPC-A1 (Fig. 2b) at both 24 h (both *P* < 0.05) and 48 h (both *P* < 0.01) after transfection. In contrast, A549 transfected with anti-miR-661 displayed slower mobility at both 24 h and 48 h after transfection (both *P* < 0.01). SPC-A1 carrying anti-miR-661 also showed similar trends at 24 h (*P* < 0.05) and 48 h (*P* < 0.01) after transfection. Transwell assay (Fig. 2c and d) also proved that miR-661 conferred stronger invasiveness to NSCLC cells (*P* = 0.006 in A549 and *P* = 0.0013 in SPC-A1) and vice versa (both *P* < 0.01). Most importantly, as in Fig. 2e and f, A549 with miR-661 overexpression produced more pulmonary nodules in nude mice than control cells through caudal vein injection (*P* < 0.0001). Above finds suggested that miR-661 regulated both proliferation and metastasis of NSCLC.

### MiR-661 modulates EMT process in NSCLC cells

**Fig. 3:**
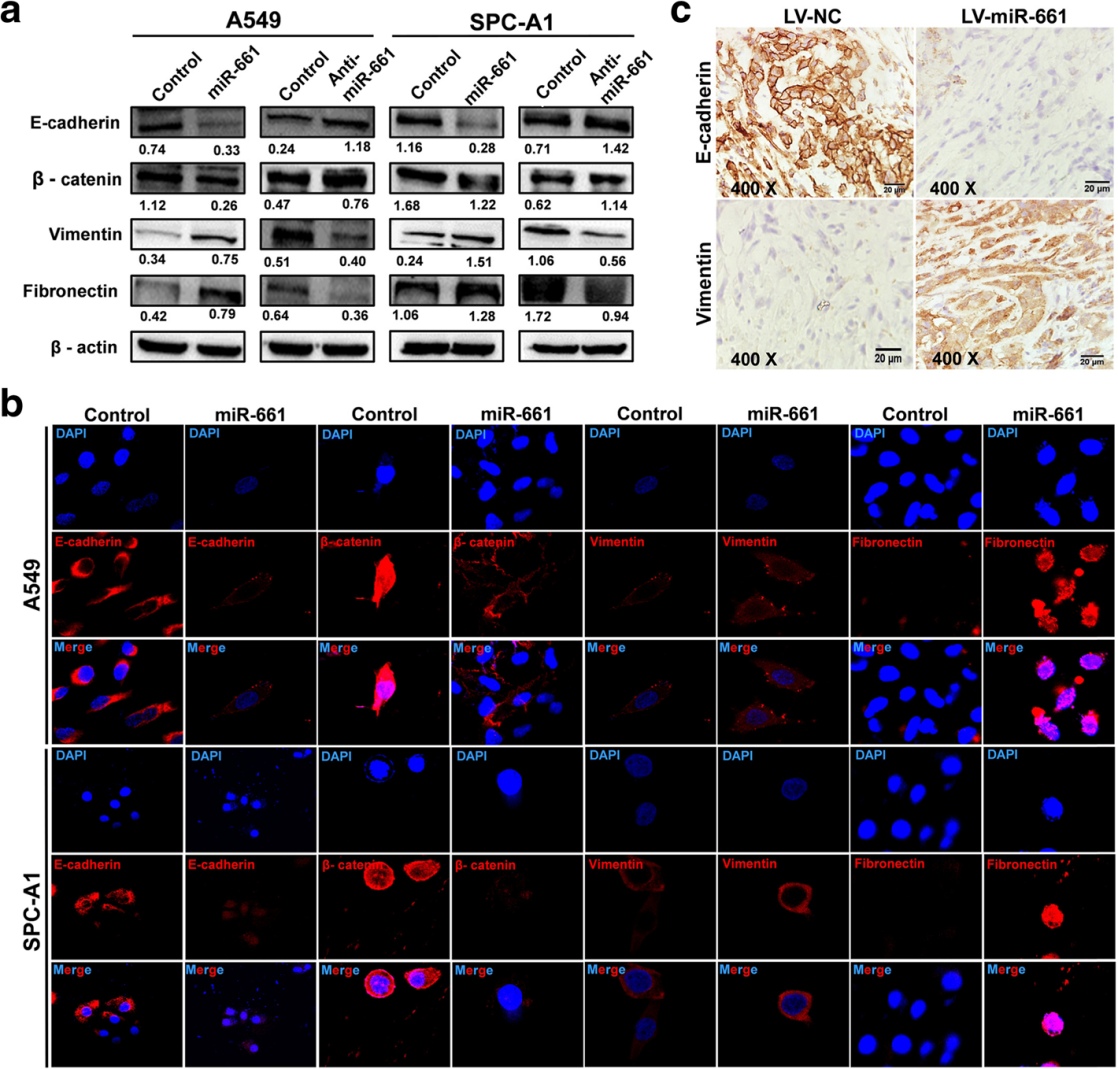
MiR-661 enhanced EMT process in NSCLC. **a**. Western blot of expression of EMT markers E-Cadherin, β-catenin, vimentin and fibronectin in A549 and SPC-A1 with miR-661 or anti-miR transfection. **b**. Immunofluorescence staining of EMT markers E-Cadherin, β-catenin, vimentin and fibronectin in A549 and SPC-A1 with miR-661 overexpression. **c**. Immunochemistry analysis of expression of epithelial marker E-cadherin and mesenchymal marker vimentin in subcutaneous tumor formed with A549 carrying LV-miR-661 or LV-NC

To determine whether miR-661 regulates metastasis of NSCLC through EMT process, we measured expression of epithelial markers E-cadherin and β-catenin as well as mesenchymal markers vimentin and fibronectin. With miR-661 overexpression (Fig. 3a), E-cadherin and β-catenin expression were remarkably decreased while vimentin and fibronectin were upregulated in both NSCLC cell lines A549 and SPC-A1. In line with these results, cell lines with anti- miR-661 transfection showed attenuation of EMT process. Immunofluorescence assay confirmed this notion by showing that overexpression of miR-661 resulted in similar trans-differentiation in both A549 and SPC-A1 (Fig. 3b). Moreover, subcutaneous tumors originated from A549 with miR-661 overexpression displayed weaker E-cadherin and stronger vimentin staining than negative controls (Fig. 3c). Thus, EMT process was activated with increased miR-661 expression in NSCLC.

### RB1 was directly targeted by miR-661 and mediated regulation on EMT and metastasis of NSCLC

**Fig. 4:**
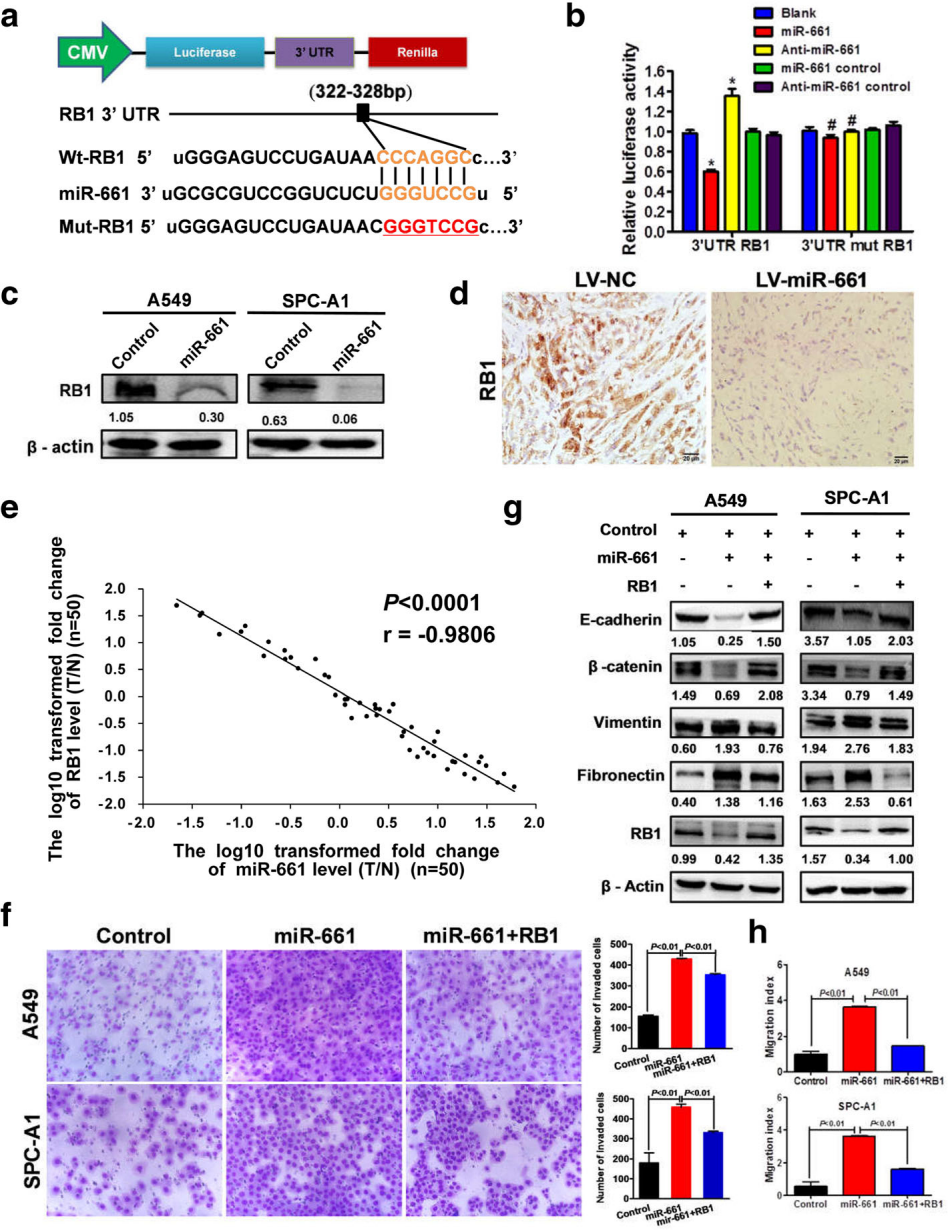
RB1 was the direct target through which miR-661 regulated EMT and invasion of NSCLC. **a**. Schematic diagram of miR-661 binding sites in the *RB1* 3′UTR. Sequences were compared between the mature miR-661 and wild-type (wt) or mutant (Mut) putative target sites in the 3′UTR of *RB1* mRNA. **b**. Relative luciferase activity of 3′UTR *RB1* and 3′UTR mut *RB1* in pre-miR-661 (miR-661) or negative control premiRNAs (Control) transfected A549 cell. Data are mean (*n* = 3) ± SEM (*, *P* < 0.01, # *P* > 0.05). **c**. Western blotting of RB1 expression in A549 and SPC-A1 cells after transient transfection with miR-661. **d**. Immunostaining of RB1 in subcutaneous tumor formed by A549 infected with LV-miR-661 or LV-NC. **e**. Correlation analysis between RB1 and miR-661 expression in 50 paired tumorous and adjacent tissues. **f**. Transwell assay of A549 and SPC-A1 with miR-661 or miR-661 + RB1 overexpression. Data are mean (*n* = 3) ± SEM. **g**. Western blotting of EMT markers in A549 and SPC-A1 with miR-661 or miR-661 + RB1 overexpression. **h**. Wound healing assay of A549 and SPC-A1 with miR-661 or miR-661 + RB1 overexpression at 48 h after transfection. Data are mean (*n* = 3) ± SEM

Database screening revealed that RB1 was a predicted target of miR-661 and there was a binding site to miR-661 in the 3′UTR of RB1 (Fig. 4a). By transiently transfected into A549 cell using a luciferase reporter vector containing the 3′UTR fragment, we found that the miR-661 instead of control miRNAs conferred reduced luciferase activity of wild-type RB1 (Wt RB1*-*3′UTR) to NSCLC cells (*P* < 0.001) (Fig. 4b), while luciferase activity of mut RB1-3′UTR was not affected by miR-661 nor control miRNAs. As in Fig. 4c, RB1 expression in A549 and SPC-A1 was downregulated upon miR-661 transfection. Subcutaneous tumor formed by A549 transducing with LV-miR-661 showed less RB1 staining (Fig. 4d), suggesting that RB1 was a direct target of miR-661.

In line with our previous findings, real-time PCR (Fig. 4e) revealed that there was a strong inverse correlation between RB1 and miR-661 expression (*r* = −0.9806, *P* < 0.0001). Restoration of RB1 voided the abatement of epithelial markers E-cadherin and β-catenin as well as augmentation of mesenchymal markers vimentin and fibronectin caused by miR-661 overexpression (Fig. 4g). Moreover, transwell assay indicated that additional RB1 abrogated the promotion on invasion of A549 and SPC-A1 (both *P* < 0.01) inducing by miR-661 (Fig. 4f). Wound healing assay also indicated that RB1 rescue caused attenuation of migrating ability of A549 and SPC-A1 overexpressing miR-661. (both *P* < 0.01). Furthermore, in vivo model confirmed above findings by showing that A549 cell with both miR-661 and RB1 overexpression yielded less pulmonary nodules than those with only elevated miR-66 l level (Additional file 2: Figure S2A–C) (*P* < 0.01). Taken together, RB1 was a direct target through which miR-661 facilitated EMT process and invasion of NSCLC.

### E2F1 interacted with RB1 and affected EMT in NSCLC

**Fig. 5:**
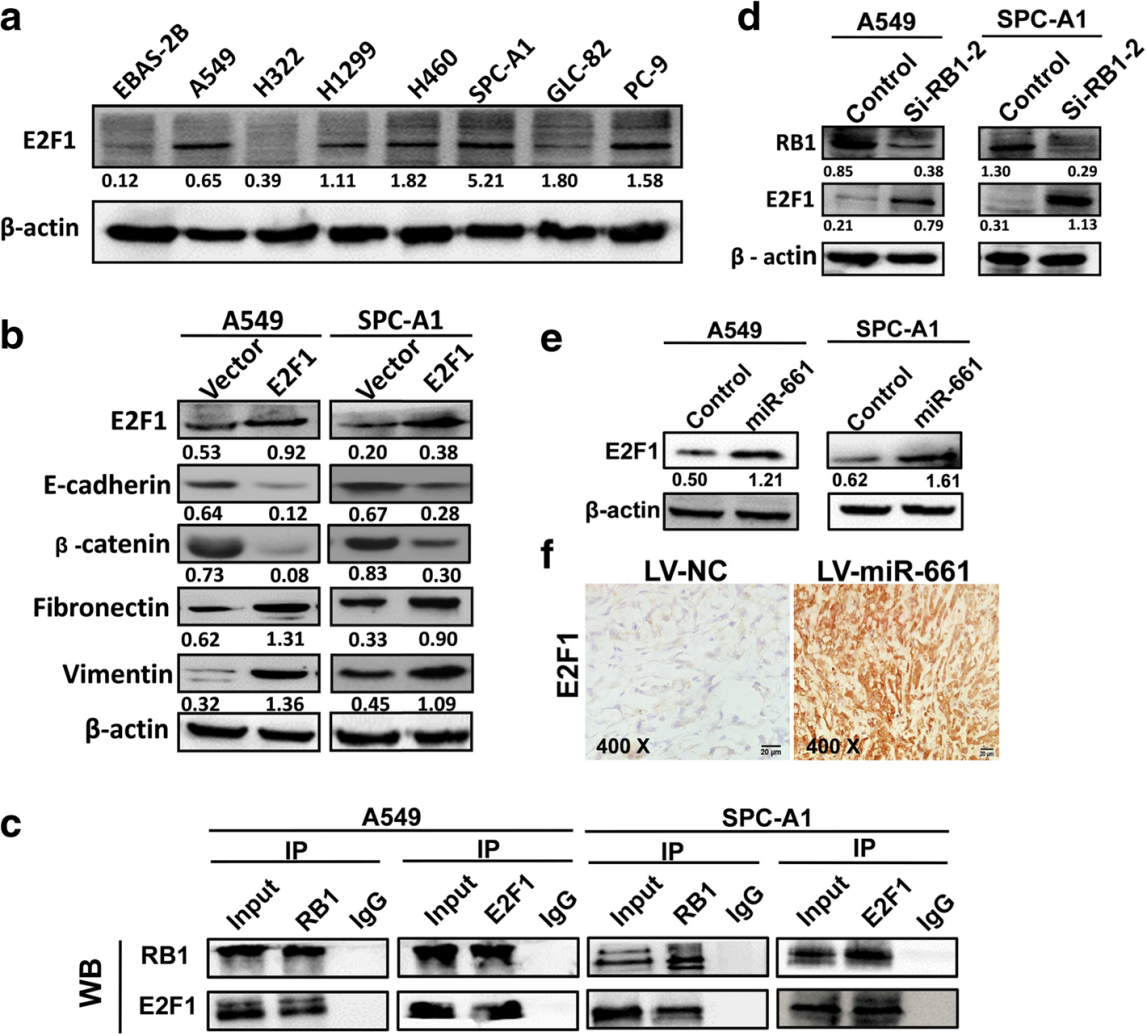
RB1 interacted with E2F1 and affected EMT process. **a**. E2F1 protein levels in NSCLC cells and human lung epithelial cells. **b**. Western blotting of EMT markers in A549 and SPC-A1 cells with E2F1 overexpression. **c**. Co-immunoprecipitation of RB1 and E2F1 in NSCLC cells. **d**. E2F1 protein levels in A549 and SPC-A1 with RB1 knockdown. **e**. E2F1 protein levels in A549 and SPC-A1 with miR-661 overexpression. **f**. Immunochemistry analysis of E2F1 expression in subcutaneous tumor formed by A549 infected with LV-miR-661 or LV-NC

E2F1 protein levels were elevated in seven NSCLC cell lines as compared with EBAS-2B (Fig. 5a), together with our findings that E2F1 upregulation increased vimentin and fibronectin while inhibited E-cadherin and β-catenin expression (Fig. 5b), supporting the view that E2F1 enhances EMT process through promoting expression of mesenchymal markers. As RB1 precipitated with E2F1 in both A549 and SPC-A1 (Fig. 5c), we postulated that RB1 could interact with E2F1 to regulate EMT. Consistently, with interference with RB1 in both cell lines led to intensified expression of E2F1 (Fig. 5d), suggesting that reduced RB1 may also regulate EMT through urging E2F1 expression. MiR-661 level was positively correlated with E2F1 expression (*r* = 0.7822, *P* < 0.0001) in human NSCLC samples (Additional file 3: Figure S3), together with the results that E2F1 were upregulated with miR-661 overexpression in vitro (Fig. 5e) and in vivo (Fig. 5f), indicating that miR-661 may be involved in invasion of NSCLC through initiation of EMT by downregulating RB1 and upregulation of E2F1.

## Discussion

Dismal metastasis is the cause of death of 90% lung cancer patients with late diagnosis [21]. Lung cancer patients reached 5 year survival rates at 50-70% if been diagnosed at early stage, while only less than 5% of those with metastatic lesion can survive under radical treatment [22]. Great progress has been made in the discovery of novel biomarkers in the past decades. Molecular markers such as EGFR, KRAS and BRAF predict patients’ survivals and response to targeted therapies [23]. Oncogenes *ALK*, *ROS1*, *RET* and *MET* have been characterized as indicators for tumor growth [24]. It is still vital to find new target to predict metastasis and develop novel modalities for prevention or treatment of NSCLC.

In this study, we observed that miR-661 expression was significantly elevated in lung cancer tissues and NSCLC cell lines, indicating that miR-661 may play a crucial role in the development of NSCLC. Interestingly, unlike its conflicting function in breast cancer [11], miR-661 expressed in lung cancer cell lines regardless of *p53* status, which implied that miR-661 may act as a tumor promoter for lung cancer in a *p53*-independent pattern. Besides, elevated miR-661 expression was correlated with shorter overall survival, suggesting that increased miR-661 level may serves as a predictor for unfavorable outcome of NSCLC patients.

Metastasis comprises complicated molecular events such as cellular adhesion reduction, cytoskeletal rearrangement, epithelial cell detachment, basal membrane impairment, stromal cell migration, colonization and proliferation [25]. MiR-661 overexpression enhanced cell viability and colonization accompanied by acceleration of G1S phase transition, indicating that miR-661 may promote proliferation of NSCLC cancer through modulating cell cycles. Moreover, in agreement with similar observations in breast cancer [10], ectopic miR-661 facilitated migration and invasion in NSCLC cells, together with our results in human NSCLC specimen, suggested that miR-661 took part in multiple steps of malignant metastasis. In vivo results that cells with increased miR-661 level grew faster and formed more lung lesions supported our view that miR-661 enhanced cell growth and invasion of NSCLC.

Epithelial-to-mesenchymal transition (EMT), a key process characterized by molecule rearrangement and cellular phenotype alternation [26, 27], is usually correlated with poor prognosis, higher metastasis rate and shorter disease free survival [28]. Loss of epithelial marker E-cadherin leads to migration of another epithelial marker β-catenin and initiates EMT [28, 29], while acquisition of mesenchymal markers vimentin and fibronectin expedite this process. Previous reports had shown that miR-661 accelerated EMT process in breast cancer cell and retinal pigmented epithelial cells [10, 30]. We found that miR-661 overexpression led to significant downregulation of epithelial markers and upregulation of mesenchymal markers, while inhibition of miR-661 yielded opposite effects in NSCLC cells A549 and SPC-A1. Thus, miR-661 may contribute to invasion and metastasis of NSCLC through furnishing both activation and acceleration of EMT process.

To further explore the mechanism involved, we identified tumor suppressor RB1 as a miR-661 target gene with application of five bioinformatics databases. Mutation or loss of function of RB1 and increased transcriptional activities of E2F1 were observed in different malignancies including NSCLC [31]. Aberrant RB1 is known to inhibit development of various cancers through modulating cell cycles, DNA replication and apoptosis [32]. But only few studies have reported the correlation between RB1 and metastasis of other cancers [33, 34]. In this study, the luciferase activity of 3′ UTR in wildtype *RB1* instead of mutant *RB1* was attenuated by miR-661 overexpression. RB1 expression of NSCLC cells were also inhibited with miR-661 overexpression. Recovery of RB1 expression partially counteracted the invasion induced by miR-661 both in vitro and in vivo, indicated RB1 as a mediator by which miR-661 regulated metastasis of NSCLC.

*RB1* knockdown induces EMT events by lessening expression of E-cadherin as well as EMT-related factors Slug and Zeb-1 in breast cancer cells [35]. Our result that RB1 restoration offset most regulation on expression of EMT markers caused by miR-661 confirmed its key role in the regulation of miR-661-induced EMT process in NSCLC. Since RB1 expression did not fully compensate the promotive effects of miR-661 on EMT, we can not rule out the possibility that miR-661 influenced other pathway to furnish EMT and metastasis of NSCLC. Furthermore, the transcriptional factor E2F1, which could bind to RB1 [36], was upregulated under RB1 downregulation in NSCLC. With RB1 interference, increased E2F1 level was observed and accompanied with enhanced expression of mesenchymal markers and attenuation of epithelial markers, implied that RB1 may hinder EMT and invasion of NSCLC via suppressing E2F1 expression. Consistently, a recent study showed that E2F1 has been implicated in regulating the mesenchymal markers of NSCLC [37]. Moreover, E2F1 expression was positively correlated with miR-661 level, supported that miR-661 may promote EMT and metastasis of NSCLC through restriction on RB1/E2F1 pathway. Other factors except for E2F1 through which RB1 downregulation by miR-661 affect EMT merits more investigation.

## Conclusions

In summary, these results suggested that miR-661 expression was correlated with cell growth, invasion and metastasis of NSCLC. RB1 was directly targeted by miR-661 and could interact with E2F1 to affect EMT process and invasion lung cancer. MiR-661 may be a novel indicator for adverse prognosis of NSCLC patients and promising target for development of new treatment.

## Additional files

Additional file 1: **Figure S1.** MiR-661 promoted cell growth of NSCLC in vitro and in vivo. A. CCK8 assays measuring effect of miR-661 on the cell proliferation of A549 and SPC-A1. B. Cell cycle analysis of A549 and SPC-A1 on miR-661 transfection. C. Colony formation assay of A549 and SPC-A1 with LV-NC or LV-miR-661 infection. D. Weights and volumes of subcutaneous tumors form by A549 infected with LV-NC or LV-miR-661. Data is mean (*n* = 3) ± SEM (* *P* < 0.05, ** *P* < 0.01). E. Ki67 staining of subcutaneous tumors formed by A549 infected with LV-NC or LV-miR-661. (PDF 1087 kb)

Additional file 2: **Figure S2.** In vivo assay of A549 with miR-661 ± RB1 expression A. Lung nodules formed after caudal vein injection of A549 carrying miR-661 ± RB1 were assessed. B. H&E staining of lung nodules formed. C. Comparison of numbers of metastatic nodules formed after caudal vein injection. All experiments were performed in triplicates. Error bars are mean (*n* = 3) ± SEM. (PDF 237 kb)

Additional file 3: **Figure S3.** Correlation analysis between E2F1 and miR-661 expression in 50 paired tumorous and adjacent tissues. (PDF 35 kb)

## Abbreviations

EMT: Epithelial-mesenchymal transition
FFPE: Formalin-fixed and parrffin-embedded
NSCLC: Non small cell lung cancer
SMUCC: Cancer Center of Southern medical university
