# Supplementary figures and images for: MiR-661 promotes tumor invasion and metastasis by directly inhibiting RB1 in non small cell lung cancer
Source: Mol Cancer. 2017 Jul 17;16:122. doi: 10.1186/s12943-017-0698-4 (PMC5514511; doi:10.1186/s12943-017-0698-4)

**A**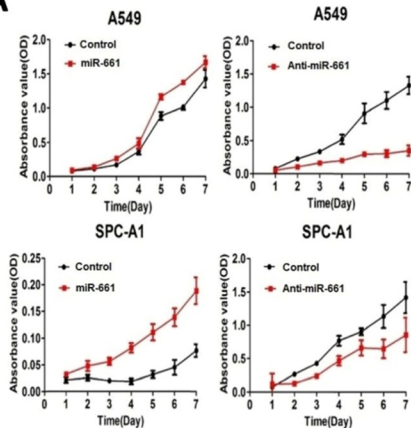**B**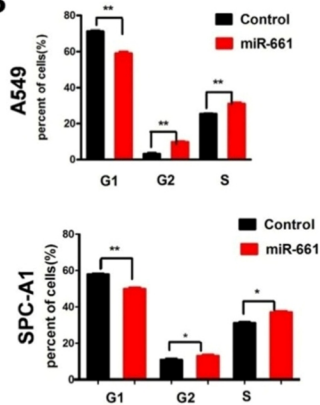**C**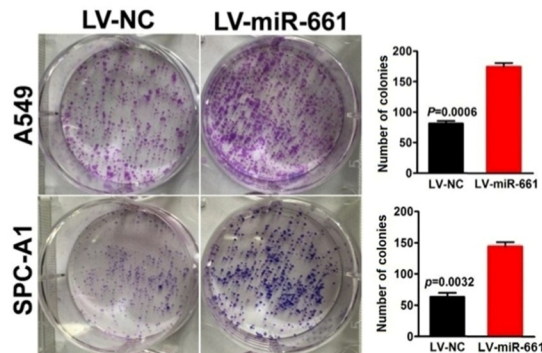**D**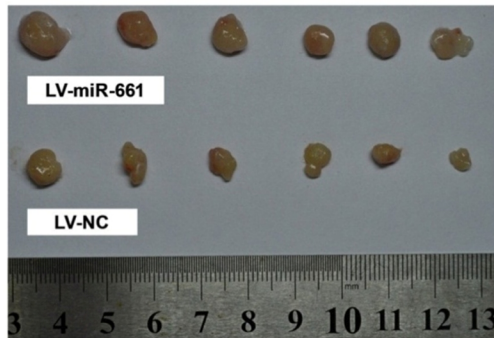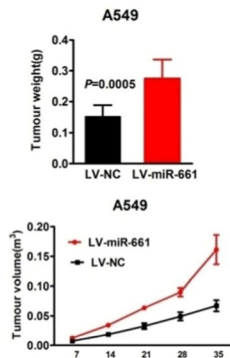**E**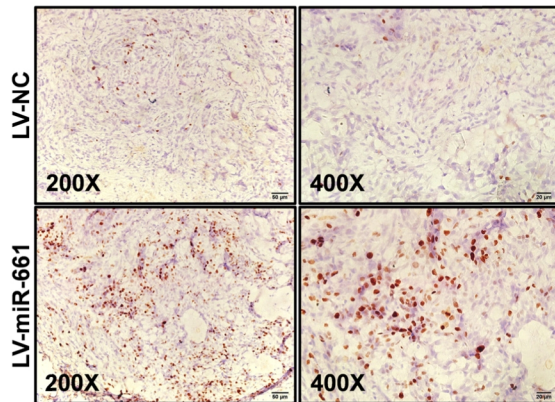

Supplement: Supplementary file 1 — Figure S1. MiR-661 promoted cell growth of NSCLC in vitro and in vivo. A. CCK8 assays measuring effect of miR-661 on the cell proliferation of A549 and SPC-A1. B. Cell cycle analysis of A549 and SPC-A1 on miR-661 transfection. C. Colony formation assay of A549 and SPC-A1 with LV-NC or LV-miR-661 infection. D. Weights and volumes of subcutaneous tumors form by A549 infected with LV-NC or LV-miR-661. Data is mean (n = 3) ± SEM (* P < 0.05, ** P < 0.01). E. Ki67 staining of subcutaneous tumors formed by A549 infected with LV-NC or LV-miR-661. (PDF 1087 kb) [file 12943_2017_698_MOESM1_ESM.pdf]

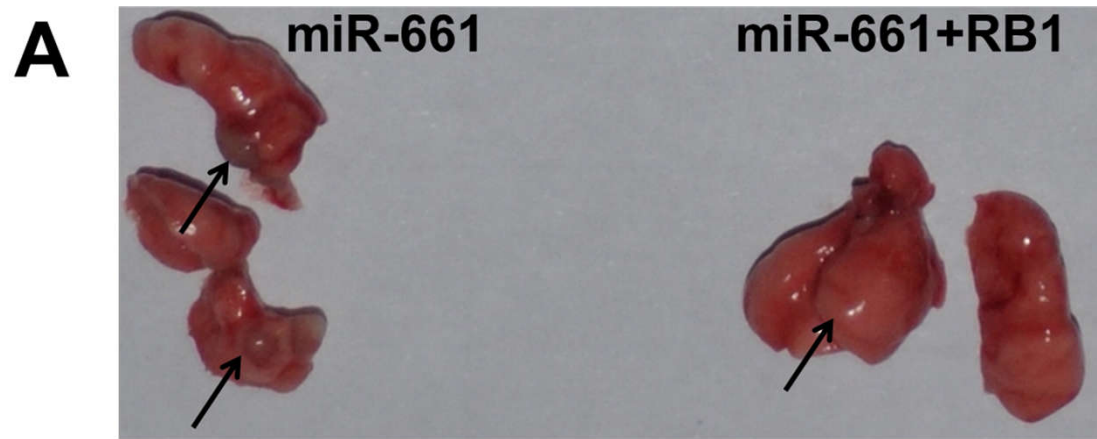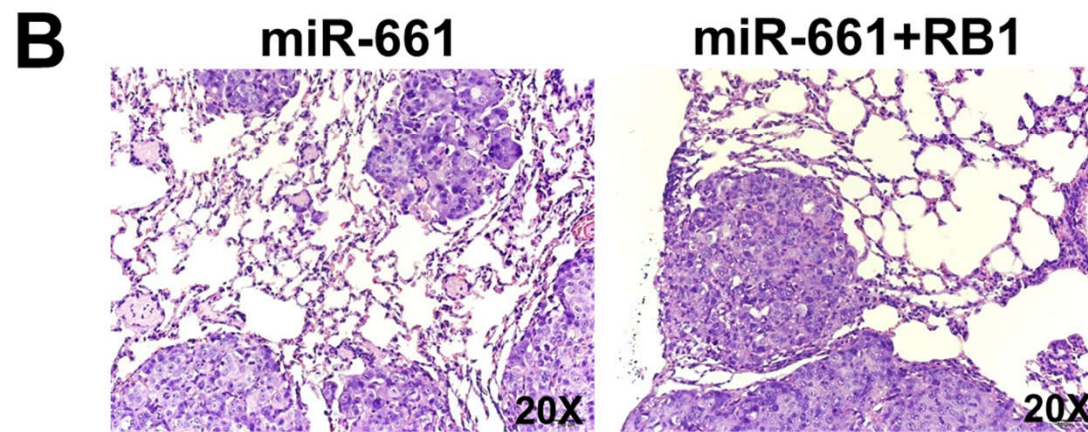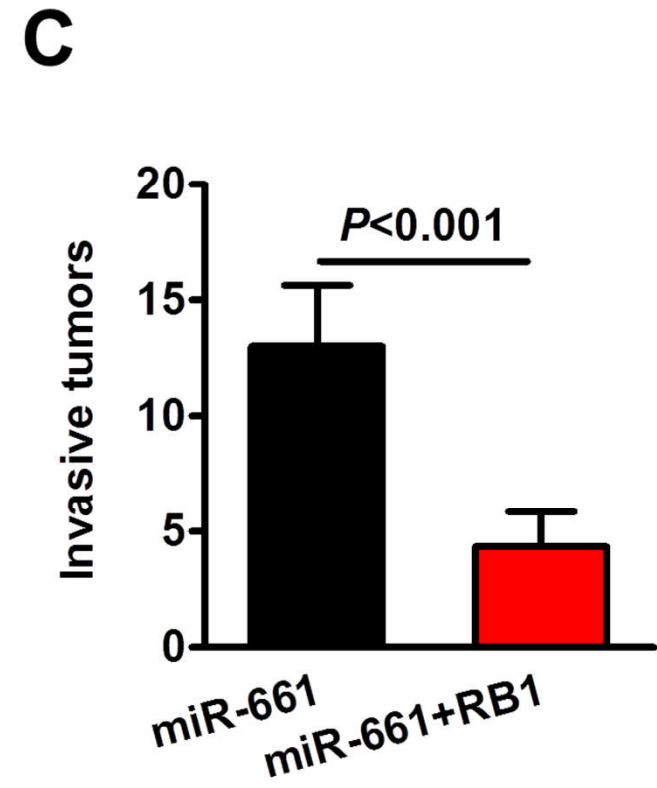

Supplement: Supplementary file 2 — Figure S2. In vivo assay of A549 with miR-661 ± RB1 expression A. Lung nodules formed after caudal vein injection of A549 carrying miR-661 ± RB1 were assessed. B. H&E staining of lung nodules formed. C. Comparison of numbers of metastatic nodules formed after caudal vein injection. All experiments were performed in triplicates. Error bars are mean (n = 3) ± SEM. (PDF 237 kb) [file 12943_2017_698_MOESM2_ESM.pdf]

The log10 transformed fold change  
Of E2F1 level (T/N) (n=50)

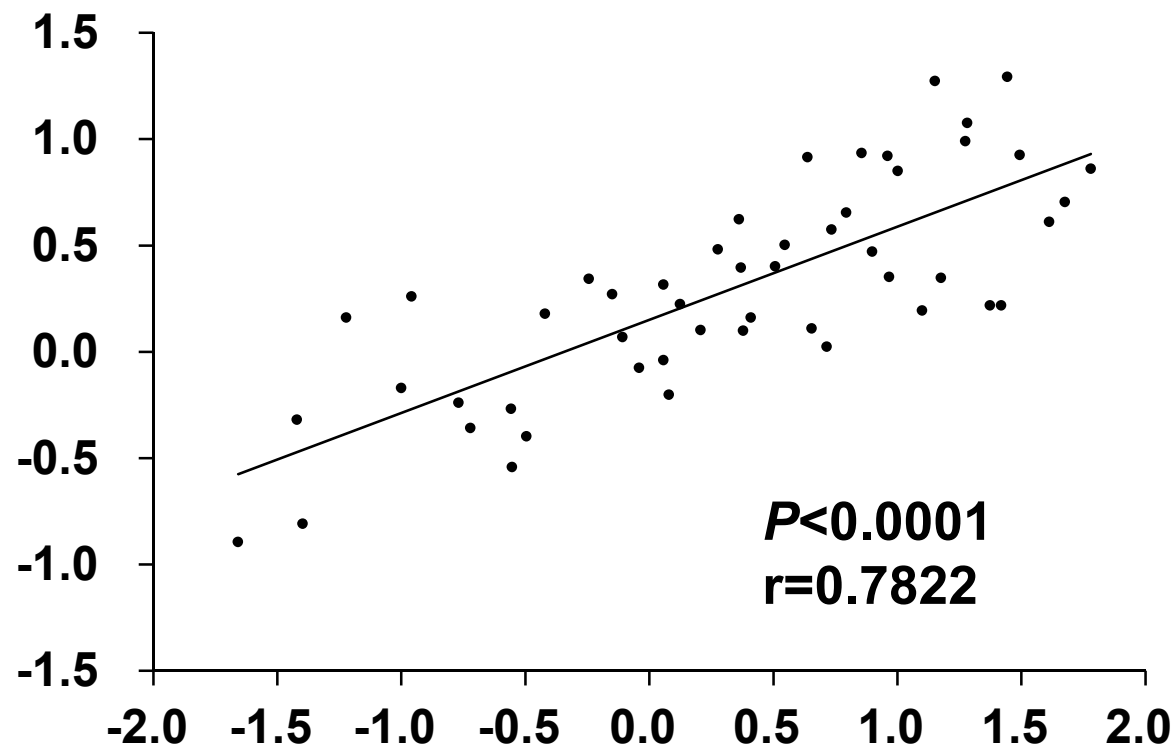

The log10 transformed fold change  
of miR-661 level (T/N) (n=50)

Supplement: Supplementary file 3 — Figure S3. Correlation analysis between E2F1 and miR-661 expression in 50 paired tumorous and adjacent tissues. (PDF 35 kb) [file 12943_2017_698_MOESM3_ESM.pdf]
